# Quantitative Determination of 18-*β*-Glycyrrhetinic Acid in HepG2 Cell Line by High Performance Liquid Chromatography Method

**DOI:** 10.1155/2018/5673186

**Published:** 2018-11-13

**Authors:** Giuseppina Nocca, Cinzia Callà, Stefano Angelo Santini, Adriana Amalfitano, Luca Marigo, Diana Valeria Rossetti, Gianrico Spagnuolo, Massimo Cordaro

**Affiliations:** ^1^Istituto di Biochimica e Biochimica Clinica, Università Cattolica del Sacro Cuore Rome, Italy; ^2^Istituto di Chimica del Riconoscimento Molecolare, CNR, c/o Università Cattolica del Sacro Cuore, L.go F. Vito 1, I-00168 Rome, Italy; ^3^UOC Chimica, Biochimica e Biologia Molecolare, Dip. Scienze di Laboratorio e Infettivologiche, Fondazione Policlinico Universitario A. Gemelli, IRCCS, Università Cattolica del Sacro Cuore Rome, Italy; ^4^UOC Odontoiatria Generale e Ortodonzia, Dip Scienze dell'Invecchiamento, Neurologiche, Ortopediche e della Testa Collo. Fondazione Policlinico Universitario A. Gemelli, IRCCS, Università Cattolica del Sacro Cuore Rome, Italy; ^5^Department of Neurosciences, Reproductive and Odontostomatological Sciences University of Naples “Federico II”, Italy; ^6^I.M. Sechenov First Moscow State Medical University, Institute of Dentistry, Moscow, Russia

## Abstract

A reverse phase high performance liquid chromatographic (RP-HPLC) method was developed for identification and estimation of 18-*β*-glycyrrhetinic acid (GA) in HepG2 cell line. The analysis was carried out using a JASCO HPLC system with a C-18 (3 *μ*m) Supelco reversed phase column (150 x 4.7 mm) using a mobile phase of 80% CH_3_OH and 20% of CH_3_CN: tetrahydrofuran: water (10:80:10, v/v/v). The method was linear in the concentration range of 1.5–120 *μ*g /mL (n = 5). The LOD and LOQ were determined based on standard deviation of the y-intercept and the slope of the calibration curve. The LOD and LOQ values were found to be 11.46 *μ*g/mL and 34.72 *μ*g/mL, respectively. The mean percentage recovery by standard addition experiments of GA is 92.4 % ± 5.2%. The intracellular GA concentration value, obtained as mean of five different determinations, was 45.8 ± 7.45 *μ*g/mL. We have developed a HPLC-UV method for quantitative determination of GA inside cells, with advantages in the cost reduction and economy of the analytical process.

## 1. Introduction

The use of natural substances as adjuvants in many drug therapies is a new important trend in modern medicine, due to a satisfactory clinical efficacy and a low degree of toxicity [1–3]. Liquorice is a perennial plant with well-known pharmacological properties that is largely employed in the cosmetic and pharmacological fields, due to its several biological effects: antimicrobial, antiulcer, immunomodulatory, anti-inflammatory, etc. [4–9].

One of the most active compounds of liquorice is the triterpenoid glycyrrhizic acid (glycyrrhizin, GZ) and the main product of its metabolism: the aglycone 18-*β*-glycyrrhetinic acid (GA) [5, 6]. GA exhibits corticosteroid and mineral-corticoid activity due to the presence of the *α*,*β*-unsaturated ketone group: in fact, GA is able to interact with mineral-corticoid and glucocorticosteroid receptors and exhibits anti-inflammatory properties [7]. Several studies [10, 11] have reported that inappropriate use of licorice can produce pseudoaldosteronism, by inactivating 11beta-hydroxysteroid-dehydrogenase [11-*β*HSD] and by binding to mineralocorticoid receptors. 11-*β*HSD catalyzes the oxidation of the active mineralocorticoid, cortisol, to the inactive cortisone and 11alpha-hydroxysteroiod-dehydrogenase is responsible for the reduction reaction. Thus, GA potentiates the anti-inflammatory activity of cortisol by inhibiting its intracellular inactivation. Moreover, GA is involved in strengthen red blood cell membrane integrity against both oxidative and proteolytic damage [12].

Moreover, in vitro studies showed that GA inhibits the proliferation of different cancer cells [13–15], without affecting normal cells [16–18]. This effect is probably due to different mechanisms, such as downregulation of glutathione (GSH) [17] and production of reactive oxygen species (ROS) [18, 19]. However, all of the above reported actions are dependent on the solubility of the compound in the hydrophilic medium and, over all, on its ability to penetrate into cells because its interactions with receptors and with enzymes occur intracellularly. Given the lipid nature of GA, in in vitro experiments, it has to be previously dissolved in DMSO [20, 21]; this solvent has a polar domain and two nonpolar groups, making GA soluble in both aqueous and organic media [22]. Moreover, DMSO can induce water pores in dipalmitoylphosphatidylcholine bilayers and this is a possible mechanism to increase penetration of active molecules through lipid membranes [23].

Several analytical methods have been developed with different techniques to evaluate the concentration of the various components of licorice in the preparations for cosmetic and biomedical application. In several publications, analytical methods have been developed using High-Performance Liquid Chromatography (HPLC) that is a very sensitive and reproducible technique [24–29]. On these bases, the aim of the present study was to develop a HPLC-UV method able to carry out a quantitative determination of GA inside HepG2 cells. This human hepatocellular cell line was chosen because these cells are able to metabolize GA [30]. To our knowledge, in this paper a method for detection of GA inside cells is reported, for the first time.

## 2. Materials and Methods

### 2.1. Chemicals and Reagents

18-*β*-glycyrrhetinic acid (GA) was purchased from Acros Organics (VWR International Srl, Milan, Italy). Cell culture medium and reagents, DMSO, ethanol (EtOH), tetrahydrofuran (HPLC grade), and acetonitrile (CH_3_CN, HPLC grade) were purchased from Sigma Chemical (Milan, Italy). Methyl alcohol (CH_3_OH, HPLC grade, Prolabo, France) and ultrapure water (obtained by a P.Nix Power System apparatus, Human, Seoul, Korea) were used for HPLC analyses.

### 2.2. HPLC Conditions

Standard solutions were analyzed using a JASCO HPLC system (2 PU-980 pumps, UV-970 UV/VIS detector and AS-1555 autosampler). The analyses were performed at a wavelength of 254 nm with a C-18 (3 *μ*m) Supelco reversed phase column (150 x 4.7 mm) using a mobile phase of 80% CH_3_OH (A) and 20% of CH_3_CN: tetrahydrofuran: water (10:80:10, v/v/v) (B) [modified from Chamoli [31] (15 min), 1.0 mL/min flow, 50 *μ*L injected volume]. Each analysis was performed five times.

### 2.3. Cell Culture

HepG2, human liver carcinoma cells (Istituto Zooprofilattico, Brescia, Italy) were grown in a 5% CO_2_ atmosphere at 37°C in IMDM (Iscove's Modified Dulbecco's Medium) with HEPES (10 mM), glucose (4.5 g/L), NaHCO_3_ (3.7 g/L), penicillin (100 units/mL), streptomycin (100 g/mL), 1% nonessential amino acids, and 10% fetal calf serum.

### 2.4. Cells Treatments

Stock solution of GA (200.0 mmol/L) was prepared immediately before use in DMSO, to obtain a final concentration of 0.1% (v/v) DMSO, which does not induce any alteration in cell vitality [32, 33]. The final concentration of GA was 200 *μ*mol/L (94.14 *μ*g/mL).

### 2.5. Determination of Intracellular Concentrations of GA

HepG2 (5 mL) were plated in 25 cm^2^ flasks at a density of approximately 25,000 cells/cm^2^ and cultured to subconfluent monolayers; GA (200 *μ*mol/L) was then added and the cells and incubated for 2 h at 37°C. Cell monolayers without GA were used as controls. After incubation, the cells were washed in PBS solution and lysed by freezing (-80°C). Cellular lysates were resuspended in 1 mL of EtOH and centrifuged (20,000 x g, 15 min, 4°C) and the supernatants were collected, evaporated, and finally resuspended in 500 *μ*L of EtOH. Samples were analyzed as reported in HPLC conditions paragraph. The concentration of GA in each sample was quantified using the calibration curve performed with standard solutions before each analysis. Each determination was repeated three times and each experiment was performed 5 times (n=5)

### 2.6. Determination of Limit of Quantitation (LOQ) and Limit of Detection (LOD)

According to the ICH recommendations [34], LOD and LOQ were determined based on standard deviation of the y-intercept and the slope of the calibration curve (n=5). Calibration curves were constructed from GA standard solutions at 5 different concentrations within the range of 1.5-120 *μ*g/mL (1.5, 15, 30, 60 and 120 *μ*g/mL).

The following equations were used for calculating LOD and LOQ:

(1) LOD=3,3×SDslopeLOQ=10×SDslope

### 2.7. Recovery Studies

The efficiency of GA recovery from cell lysates was evaluated adding two different concentrations (20 and 40 *µ*g/mL) into control cellular lysates. Two replicates of each concentration were prepared for each lysate. The absolute recovery was evaluated as the ratio between the experimentally observed concentration and the theoretical concentration.

## 3. Results and Discussion

GA is a compound with several biological activities; for this reason, it is widely used both in cosmetic and in medical preparations. Since the biological effects of GA depend on its intracellular concentration and not only its concentration in culture medium, a simple HPLC-UV method for the intracellular GA determination was developed and described in this paper.

To be sure to be able to detect the exact intracellular GA concentration, the method was first tested for linearity, using calibrating standard solution mixtures of the GA in concentration range 1.5–120 *μ*g/mL. Each determination was repeated three times and each calibration curve was performed 5 times (n=5). The obtained regression equation was Y = 22017*∗*X – 21465 with* R*^2^ = 0.9946, confirming the linearity of the response under the conditions used. The LOD and LOQ were determined based on standard deviation of the y-intercept and the slope of the calibration curve. The LOD and LOQ values were found to be 11.46 *μ*g/mL and 34.72 *μ*g/mL, respectively.

We were then able to apply our analytical method to identify and quantify the intracellular GA in cells treated with a concentration of 200 *μ*mol/L of GA in cell culture medium (94.14 *μ*g/mL; 5 mL). In these experimental conditions, no cytotoxic effects were observed after two hours (data not shown). In this preliminary phase of the study, this is very important, since intracellular concentration of GA could affect cellular vitality.

**Figure 1:**
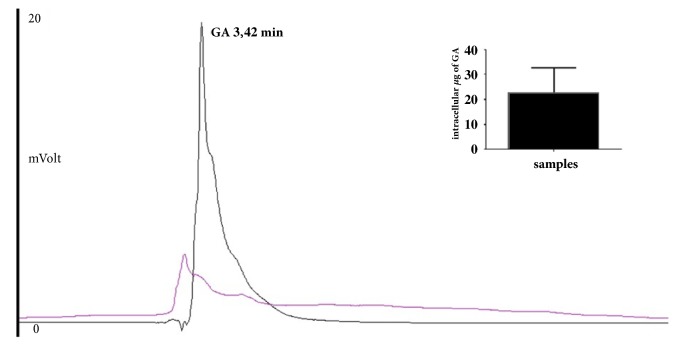
Chromatographic profiles of HepG2 lysates. In intracellular sample, incubated for 2h with GA, a signal that corresponds to a substance with the same elution time of GA was found (black); the described signal was not present in the untreated samples (pink). Inset: Intracellular GA amount.

As shown in Figure 1 a signal corresponding to a substance with the same elution time of GA was found in cellular lysates after incubation for 2 h with GA as shown in materials and methods; the described signal was not present in the untreated samples (controls).

The intracellular GA concentration value, obtained as mean of five different experiments, was 45.8 ± 7.45 *μ*g/mL. Considering that the final volume of samples resuspended in EtOH was 0.5 mL (see materials and method) the true amount of GA inside the cells was about 23 *μ*g (Figure 1, insert).

The GA percentage mean recovery by standard addition experiments is 92.4%  ±  5.2% (data not shown). These results indicate the reliability of our analytical method.

## 4. Conclusions

Our HPLC-UV method for identification and quantification of GA inside HepG2 cells, reliable in linearity and recovery, could be used in different fields of pharmacology research where the measurement of intracellular concentrations of GA is mandatory.

## Data Availability

The datasets generated during and/or analysed during the current study are available from the corresponding author on reasonable request.
